# Are the class 18 myosins Myo18A and Myo18B specialist sarcomeric proteins?

**DOI:** 10.3389/fphys.2024.1401717

**Published:** 2024-05-09

**Authors:** Markus Horsthemke, Charles-Adrien Arnaud, Peter J. Hanley

**Affiliations:** ^1^ IMM Institute for Molecular Medicine, HMU Health and Medical University Potsdam, Potsdam, Germany; ^2^ Department of Medicine, Science Faculty, MSB Medical School Berlin, Berlin, Germany

**Keywords:** unconventional myosins, MYO18A, MYO18B, sarcomere, stress fibers, knockout (KO) mice

## Abstract

Initially, the two members of class 18 myosins, Myo18A and Myo18B, appeared to exhibit highly divergent functions, complicating the assignment of class-specific functions. However, the identification of a striated muscle-specific isoform of Myo18A, Myo18Aγ, suggests that class 18 myosins may have evolved to complement the functions of conventional class 2 myosins in sarcomeres. Indeed, both genes, *Myo18a* and *Myo18b*, are predominantly expressed in the heart and somites, precursors of skeletal muscle, of developing mouse embryos. Genetic deletion of either gene in mice is embryonic lethal and is associated with the disorganization of cardiac sarcomeres. Moreover, Myo18Aγ and Myo18B localize to sarcomeric A-bands, albeit the motor (head) domains of these unconventional myosins have been both deduced and biochemically demonstrated to exhibit negligible ATPase activity, a hallmark of motor proteins. Instead, Myo18Aγ and Myo18B presumably coassemble with thick filaments and provide structural integrity and/or internal resistance through interactions with F-actin and/or other proteins. In addition, Myo18Aγ and Myo18B may play distinct roles in the assembly of myofibrils, which may arise from actin stress fibers containing the α-isoform of Myo18A, Myo18Aα. The β-isoform of Myo18A, Myo18Aβ, is similar to Myo18Aα, except that it lacks the N-terminal extension, and may serve as a negative regulator through heterodimerization with either Myo18Aα or Myo18Aγ. In this review, we contend that Myo18Aγ and Myo18B are essential for myofibril structure and function in striated muscle cells, while α- and β-isoforms of Myo18A play diverse roles in nonmuscle cells.

## Introduction

In mouse and human, the myosin superfamily of motor proteins is divided into 12 classes. Conventional class 2 myosins have long coiled-coil domains which mediate dimerization and assembly into bipolar (thick) filaments, whereas the other 11 classes are collectively referred to as unconventional myosins and serve various specific roles (Kalhammer and Bähler, 2000; Sellers, 2000; Hartman et al., 2011; Maravillas-Montero and Santos-Argumedo, 2012; Batters and Veigel, 2016; Coluccio, 2020). All myosins contain a conserved head (motor) domain which typically exhibits ATPase activity and binds to filamentous actin (F-actin), enabling the chemical energy from ATP hydrolysis to be converted to force production (Mermall et al., 1998; Sellers, 2000). Class 18 myosins are encoded by two genes: *Myo18a* and *Myo18b* in mice, and *MYO18A* and *MYO18B* in humans (Bruford et al., 2020). Extensive biochemical analyses of purified myosin 18A (Myo18A) and myosin 18B (Myo18B) proteins or protein fragments have revealed, surprisingly, that these myosins exhibit negligible ATPase activity (Guzik-Lendrum et al., 2013; Taft et al., 2013; Latham et al., 2020; Taft and Latham, 2020), perhaps barely sufficient to justify membership in the myosin superfamily. Nevertheless, class 18 myosins have strong phylogenetic roots and show localization to actin-based structures, such as actin stress fibers and sarcomeres (Salamon et al., 2003; Mori et al., 2005; Ajima et al., 2008; Billington et al., 2015; Horsthemke et al., 2019; Latham et al., 2020; Taft and Latham, 2020; Ouyang et al., 2021). Whether Myo18A and Myo18B exhibit ATPase activity in their natural environment remains to be clarified. Alternatively, these proteins may act as scaffold proteins or inhibitors (brakes) to actin translation by bipolar myosin two filaments, among other functions. In this review, we contend that Myo18B and the gamma-isoform of Myo18A, Myo18Aγ, have evolved as indispensible structural elements or regulators of sarcomeres. We also discuss the diverse functions ascribed to the PDZ-containing alpha-isoform of Myo18A, Myo18Aα, and we briefly speculate on the roles of the beta-isoform, Myo18Aβ.

### Myosin 18A

Myo18A was first identified over two decades ago (Furusawa et al., 2000). In 2000, Furusawa et al. (Furusawa et al., 2000) cloned a novel myosin, which was initially denoted MysPDZ (myosin containing PDZ) since it harbors an N-terminal extension containing a PDZ domain in addition to a KE (lysine and glutamic acid)-rich sequence. Northern blot analysis revealed that MysPDZ (7.5 kb transcript), now known as Myo18Aα, is widely expressed in mouse tissues, whereas a 7.0 kb transcript is additionally expressed in hematopoietic cells and a 10.5 kb transcript is expressed in heart and skeletal muscle. In further work, Mori et al. (Mori et al., 2003) showed that the 7.0 kb transcript corresponds to an isoform lacking the PDZ-containing N-terminal extension, which was denoted MysPDZβ, as distinct from MysPDZα (MysPDZ), and is now denoted Myo18Aβ. Fluorescence imaging of either Myo18A labeled with antibodies against the coiled-coil domain or expressed YFP-tagged Myo18A constructs revealed that MysPDZα (Myo18Aα) localizes to the perinuclear region, possibly corresponding to the endoplasmic reticulum and Golgi apparatus, and the actin cytoskeleton, whereas MysPDZβ (Myo18Aβ) localizes diffusely in the cytoplasm. Truncation mutants fused to YFP or a Myc tag (also known as c-Myc tag) indicated that the KE-rich sequence was required for localization to F-actin, whereas the PDZ domain mediated localization to the plasma membrane (Mori et al., 2005).

The physiological roles of the widely expressed PDZ-containing myosin Myo18Aα have not yet been conclusively established. PDZ domains typically function as scaffolding modules (molecular glue), mediating protein-protein interactions and often localizing proteins to specific subcellular locations (Bezprozvanny and Maximov, 2001; Liu and Fuentes, 2019). These domains recognize short peptide motifs at the C-terminus of their target proteins, which include membrane-bound receptors and ion channels. Using anti-surfactant protein A receptor 210 antibodies, affinity chromatography, and mass spectrometry, Yang et al. (Yang et al., 2005) deduced that Myo18A is the molecular correlate of surfactant protein A receptor 210, which mediates the clearance of pathogens opsonized with surfactant protein A, a collectin secreted by alveolar epithelial type II cells (Casals et al., 2018; King and Chen, 2020). The authors identified a putative transmembrane α-helix in the head domain of Myo18A, suggesting that it is a single-pass membrane protein. Moreover, antibodies targeted to the neck domain of Myo18A blocked the binding of surfactant protein A to macrophages, implying that the N-terminus is on the cytosolic side of the plasma membrane. The apparent minimal ATPase activity of Myo18A (Guzik-Lendrum et al., 2013; Taft et al., 2013) would support a potential role of Myo18A as a membrane-bound receptor. Macrophages predominantly express Myo18Aβ (Horsthemke et al., 2019) which would be expected to bind surfactant protein A without further action, whereas expression of Myo18Aα, which occurs, for example, following activation of macrophages with leukemia inhibitory factor (Mori et al., 2003), is probably required to recruit proteins via its cytosolic PDZ domain and initiate phagocytosis. More recently, macrophages isolated from myeloid-restricted *Myo18a* conditional knockout mice were shown to exhibit normal motility, chemotaxis, and phagocytosis, but, unfortunately, surfactant protein A binding or phagocytosis mediated by surfactant protein A opsonization were not investigated (Horsthemke et al., 2019). However, RAW 264.7 cells (macrophage cell line) stably transfected with a dominantly negative truncated Myo18Aα mutant exhibited markedly impaired uptake of surfactant protein A-opsonized *Staphylococcus aureus* (Sever-Chroneos et al., 2011). On the contrary, the diffuse cytosolic localization of N-terminally YFP-tagged Myo18Aβ (Mori et al., 2003), which corresponds to the short variant of surfactant protein A receptor 210 (Yang et al., 2005), argues against a role for Myo18A isoforms as plasma membrane receptors. Lack of membrane localization of YFP-Myo18Aβ cannot be explained by an inhibitory effect of the fluorescent protein tag since N-terminally YFP-tagged Myo18Aα localizes to the plasma membrane (Mori et al., 2003). Further investigations are necessary to confirm whether Myo18A is indeed a transmembrane protein which binds surfactant protein A-opsonized particles. However, it is most unlikely that the Myo18A head domain spans the plasma membrane since this would require extensive unfolding of this highly conserved structure.

In 2009, Dippold et al. (2009) reported that Myo18A, identified by co-immunoprecipitation and mass spectrometry, is a binding partner of GOLPH3 (Golgi phosphoprotein 3). When Myo18A was knocked down in HeLa cells using siRNA, it mimicked the effects of GOLPH3 knockdown by inducing a more condensed (less stretched) Golgi structure and reducing vesicle budding. Expression of GFP-tagged mouse Myo18A, predicted to be resistant to the siRNA used to target human Myo18A, rescued the Golgi morphology phenotype, whereas a motor mutant (lacking ATPase activity) failed to rescue the phenotype. These data implied a model in which Myo18A is linked to Golgi via GOLPH3 and binds to actin filaments to exert force (actomyosin-ATPase activity) and produce a flattened stack of cisternae, the characteristic morphology of the Golgi apparatus. At variance with this model, two independent groups showed that Myo18A binds weakly to F-actin, but only exhibits negligible ATPase activity, even in the presence of GOLPH3 (Guzik-Lendrum et al., 2013; Taft et al., 2013; Bruun et al., 2017). Indeed, *in vitro* gliding assays revealed that Myo18A inhibits the translocation of actin filaments by class 2 myosin (Guzik-Lendrum et al., 2013). However, Taft et al. (2013) showed that GOLPH3 interacts with the PDZ domain of Myo18A and increases its affinity for actin. One possibility to explain the incongruent findings is that mixed bipolar filaments containing both nonmuscle myosin 2 and Myo18Aα (Billington et al., 2015) could provide a means for stretching Golgi cisternae. In this scenario, nonmuscle myosin 2 exerts force and Myo18Aα binds to GOLPH3 and acts as an F-actin tethering protein (Guzik-Lendrum et al., 2013; Taft et al., 2013), along the lines schematically illustrated by Billington et al. (2015). Bruun et al. (2017) re-investigated the role of Myo18A in Golgi morphology and found using two different anti-Myo18A C-terminus antibodies that endogenous Myo18A, as well as GFP-tagged Myo18Aα, did not localize to Golgi. The authors also showed that knockdown of Myo18A using shRNA did not affect Golgi morphology. Similarly, Horsthemke et al. (2019) observed no differences in the Golgi morphology of resident peritoneal macrophages isolated from wild-type and myeloid-restricted *Myo18a* conditional knockout mice. Thus, although Myo18A interacts with Golgi proteins, the extent and functional importance of Myo18A localization to Golgi remains to be clarified.

Various studies have implicated Myo18A in cell motility. Tan et al. (2008) deduced that Myo18A is part of a tripartite protein complex essential for cell motility. This complex, which also includes LRAP35a (leucine repeat adapter protein 35a), encoded by *LURAP1* and also known as leucine rich adaptor protein 1, and MRCK (myotonic dystrophy kinase-related Cdc42-binding kinase), encoded by *CDC42BPA* and also known as CDC42-binding protein kinase alpha, promotes nonmuscle myosin 2-dependent actomyosin assembly and retrograde actomyosin flow. The authors stressed that the Myo18A-containing tripartite protein complex localizes to retrograde moving actomyosin bundles in the lamella, which consist of nonmuscle myosin 2-rich actin arcs and dorsal stress fibers (Burnette et al., 2014), but not actin stress fibers, also known as ventral stress fibers or subnuclear actin stress fibers, which contain nonmuscle myosin 2 and are anchored to the substrate at each end by focal adhesions. siRNA-mediated knockdown of Myo18A led to loss of actomyosin structures in the lamella. Moreover, MRCK localization to the lamella was decreased by either knockdown of Myo18A or introduction of a motor mutant (lacking ATPase activity) of Myo18A. Using U2OS cells (human epithelial cell line derived from an osteosarcoma) and wound-healing assays, Tan et al. (2008) also showed that microinjection of a dominant-negative construct of Myo18A inhibited cell migration. These data suggest that Myo18A is required for the formation and/or maintenance of nonmuscle myosin 2-containing actin arcs and dorsal stress fibers. In accord with Tan et al. (2008), Billington et al. (2015) clearly showed using confocal microscopy that GFP-tagged Myo18Aα and GFP-tagged Myo18Aβ colocalized with tdTomato-tagged nonmuscle myosin 2A in lamellar protrusions of U2OS cells, but GFP-tagged Myo18Aα additionally localized to subnuclear actin stress fibers. The authors also showed by co-sedimentation and electron microscopy that polymerization of nonmuscle myosin 2A with Myo18Aβ produced mixed bipolar filaments. Indeed, mixed bipolar filaments containing nonmuscle myosin 2A together with Myo18Aα or Myo18Aβ could be resolved in cells using superresolution imaging, obtained by TIRF-SIM (combined total internal reflection fluorescence (TIRF) and structured illumination microscopy (SIM)). Thus, imaging by various independent groups strongly supports the notion that Myo18Aα associates with nonmuscle myosin 2-containing stress fibers, but there are conflicting results in relation to Myo18Aβ (Mori et al., 2005; Billington et al., 2015). We assume that the N-terminal extension of Myo18Aα, lacking in Myo18Aβ, is required for localization to actin stress structures. The role of Myo18Aβ remains unclear, although it has been shown to form antiparallel dimers in high-salt buffer (Billington et al., 2015) and may heterodimerize with Myo18Aα. Further work is required to clarify the subcellular localization and function of Myo18Aβ.

Myo18A was also implicated in regulating actin cytoskeletal dynamics and cell motility by Hsu et al. (2010), who identified Myo18A as an interaction partner of PAK2 (p21-activated kinase 2) by co-immunoprecipitation and mass spectrometry. PAKs are a family (PAK1–PAK6) of serine/threonine kinases which interact with and are thought to be important downstream targets of the p21 Rho GTPases Rac1 and Cdc42, each of which induce actin polymerization and membrane protrusions and are key mediators of cell motility and chemotactic navigation (Lawson and Ridley, 2018). The authors deduced that PAK2 binds to Myo18A via the βPIX/GIT1 (β-PAK-interacting exchange factor and G protein-coupled receptor kinases interactor 1) complex. βPIX, encoded by *ARHGEF7* (Rho guanine nucleotide exchange factor 7), interacts with group I PAKs (PAK1–PAK3) though its N-terminal SH3 (Src homology 3) domain. This interaction leads to the activation of Rac and Cdc42 via the Dbl-homology (DH) domain of βPIX, which serves as a selective GEF (guanine nucleotide exchange factor) for Rac and Cdc42. Following guanine nucleotide exchange, Rac-GTP and Cdc42-GTP activate group I PAKs, which in turn inhibit cofilin via LIM kinases, among other functions. Thus, βPIX not only activates Rac and Cdc42 but also serves as a link to their downstream targets (PAK1–PAK3). Hsu et al. (2010) showed that knockdown of Myo18A did not impair the formation of PAK/βPIX/GIT1 complexes, but induced morphological changes, including marked cell spreading and a reduction in dorsal ruffles, as well as decreased cell migration in wound healing assays. Truncation mutant analysis indicated that the C-terminus of Myo18Aα, also present in Myo18Aβ, interacts with βPIX. In complementary work, Hsu et al. (2014) showed that deletion of the C-terminal extension impairs cellular localization of βPIX in A431 cells and decreases cell motility. Consistent with these findings, Myo18Aα was shown to target the Rac-/Cdc42-GEF βPIX to the dendritic spines of cultured Purkinje neurons, whereas knockdown of Myo18Aα or deletion of the C-terminal Myo18Aα-binding site of βPIX markedly decreased βPIX enrichment in spines, which was associated with loss of F-actin and nonmuscle myosin 2 in these structures (Alexander et al., 2021). Thus, Myo18Aα and possibly also Myo18Aβ localize to nonmuscle myosin 2-containing stress fibers and interact with proteins that regulate actin dynamics. To gain further insight, phenotypic analysis of mice selectively lacking both Myo18A isoforms would be most helpful, especially if homozygous mutants prove to be viable.

In developing zebrafish embryos, *myo18a* genes, *myo18aa* and *myo18ab*, were observed to be expressed in the somites (Cao et al., 2014). Knockdown of either gene resulted in mildly irregular localization of dystrophin and α-dystroglycan, two key proteins involved in linking F-actin to the extracellular matrix. This irregularity manifested as unsharp somite demarcations and was associated with disrupted myofibers (skeletal muscle cells), identified using anti-slow myosin heavy chain antibodies. Simultaneous knockdown of both genes or overexpression of the PDZ-containing N-terminal extension resulted in a more pronounced phenotype. Further work by Cao et al. (2016) showed that Myo18A is required for adhesion of cultured zebrafish embryo myoblasts to laminin-coated substrates. Together with other findings, including interaction of Myo18A with the Golgi protein Golgin45, Cao et al. (2016) deduced that the N-terminal extension of Myo18A acts as a scaffold to bind various signaling molecules, link Golgi to F-actin, and stabilize myoblast adhesion to the extracellular matrix.

**FIGURE 1:**
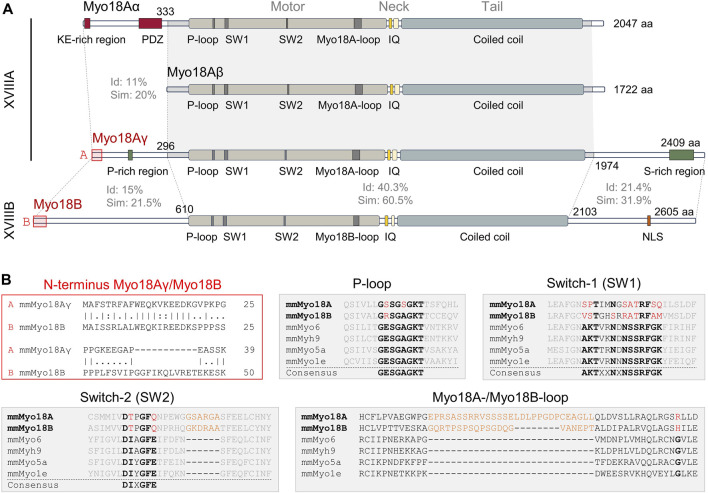
Domain structures and sequence alignments of Myo18A and Myo18B. **(A)**, Domain structures of mouse (*Mus musculus*) Myo18Aα, Myo18Aβ, Myo18Aγ, and Myo18B. Sequence identity (Id) and similarity (Sim) are indicated for various regions. Abbreviations: KE-rich, lysine (K) and glutamic acid (E)-rich; PDZ, post-synaptic density (PSD) protein, *Drosophila* discs-large (Dlg) tumor suppressor protein, and zonula occludens (ZO) protein; P-loop, phosphate-binding loop; SW1, switch-1; SW2, switch-2; IQ, IQ (isoleucine (I) and glutamine (Q)) calmodulin-binding motif; aa, amino acids; P-rich, proline (P)-rich; S-rich, serine (S)-rich; NLS, nuclear localization signal. **(B)**, Alignment of the extreme N-terminal ends of Myo18Aγ and Myo18B (marked by red boxes in panel **(A)**, highlighting high identity and similarity. In the aligned sequences, vertical lines (“|”) indicate positions of identity (amino acids are identical), colons (“:”) indicate positions of similarity (amino acids have similar side chain properties), single dots (“.”) indicate mismatch of amino acids, and dashes (“–”) indicate gaps in a sequence relative to its counterpart. The gray filled boxes show alignment of *Mus musculus* (*Ms*) Myo18A and *Ms* Myo18B P-loop, switch-1, and switch-2 sequences with those of *Ms* Myo6, *Ms* Myh9 (nonmuscle myosin IIA), *Ms* Myo5a, and *Ms* Myo1e. Conserved sequence motifs are highlighted in bold black, while deviations in Myo18A and Myo18B are shown in red. Inserts specific for Myo18A and Myo18B are shown in orange.

Deletion of *Myo18a* in mouse is embryonic lethal at around embryonic day 12.5, but surprisingly lacZ (X-Gal) staining indicated that *Myo18a* is highly expressed in the developing heart, as well as in somites (Horsthemke et al., 2019). Cardiac myocyte-restricted deletion of *Myo18a* in mice was similarly embryonic lethal and electron microscopy revealed disorganized cardiac sarcomeres in embryos carrying homozygous *Myo18a* mutations. More surprisingly, a novel isoform of Myo18A, denoted Myo18Aγ, was detected in the heart which was larger than Myo18Aα and contained alternative N- and C-terminal extensions (Horsthemke et al., 2019). Notably, the N-terminal extension of Myo18Aγ does not contain either a KE-rich sequence or PDZ domain, but instead contains a polyproline helix (Figure 1A). Myo18Aγ-GFP expressed in neonatal rat ventricular myocytes clearly showed localization to the sarcomeric A-band. All of these findings are reminiscent of Myo18B, discussed in the next section, and suggest that each class 18 myosin gene encodes a protein required for sarcomere function.

Most recently, Myo18Aα was shown to immunoprecipitate with GIPC3 (GIPC PDZ domain containing family, member 3) and the authors showed that the PDZ domain of GIPC3 interacts with the C-terminus of Myo18Aα, which is shared by Myo18Aβ (Chatterjee et al., 2023). GIPC3 localizes to the cuticular plate of inner and outer hair cells of the cochlea during postnatal development. Each stereocilium of the hair cells inserts into the cuticular plate, a dense network of filamentous actin. SIM imaging of immunolabeled Myo18Aα showed that it localizes immediately below the actin-rich cuticular plate. Mutations of various unconventional myosin genes are associated with deafness (Friedman et al., 1999; Moreland and Bird, 2022), including *Myo6* (Avraham et al., 1995), *Myo7a* (Gibson et al., 1995; Weil et al., 1995), and *Myo15a* (Wakabayashi et al., 1998; Liang et al., 1999; Belyantseva et al., 2003). Whether mutations or deletion of *Myo18a* in hair cells causes significant abnormalities in the morphology and function of hair cells remains to be determined.

### Myosin 18B

In a human squamous cell lung carcinoma cell line, Lu24, a homozygous deletion on chromosome 22q12.1 suggested the presence of a tumor suppressor gene within the deletion, ultimately leading to the identification of a novel myosin gene, denoted *MYO18B*, which is structurally related to *MYO18A* (Nishioka et al., 2002). Consistent with a potential role as a tumor suppressor gene, *MYO18B* is inactivated by deletions, mutations, or methylation in about 50% of lung carcinomas (Yokota et al., 2003). Further studies have shown that MYO18B expression is reduced in various cancers, including primary ovarian and colorectal carcinoma, and restoration of MYO18B expression in pleural mesothelioma cell lines decreased tumor growth and metastatic potential. In contrast, high MYO18B expression was correlated with poor prognosis in hepatocellular carcinoma and knockdown of *MYO18B* in HepG2 cells (human hepatocellular carcinoma cell line) decreased cell proliferation and invasiveness. Thus, MYO18B has been associated with either tumor suppression or progression and the role of MYO18B presumably varies depending on the specific cancer type and the cellular context. Interestingly, among prostate cancer cell lines, *MYO18A* expression was shown to be higher in a cell line with higher metastatic potential, and the authors inferred, based on knockdown studies, that effects of MYO18A on actin organization and motility could contribute to metastasis (Makowska et al., 2015).

Nishioka et al. (2002) also investigated the expression of *MYO18B* across various human tissues. Northern blot analysis revealed that MYO18B transcripts (∼8 kb) were expressed in heart and skeletal muscle, but not in other tissues. Using real-time quantitative PCR, which is much more sensitive than Northern blot, *MYO18B* expression was detected in a broader range of tissues, including bone marrow, thymus, and testis. Phylogenetic analysis highlighted that MYO18A genes are expressed in both vertebrates and invertebrates, whereas human and mouse Myo18B genes appear to have arisen from a duplication in vertebrates. Using human and mouse tissues, Salamon et al. (2003) similarly could only detect MYO18B mRNA in heart and skeletal muscle using Northern blotting, with higher levels in the heart. RT-PCR corroborated these findings, but expression of MYO18B mRNA could be detected in other tissues, albeit after a high number of PCR cycles. The authors showed that *Myo18b* expression is weak in C2C12 cells (mouse myoblast cell line), but it increases following induction of myogenesis (myogenic differentiation), reaching a peak and leveling off at around day 3 after induction. When cells were stably transfected with Myo18B-Myc, the tagged protein was found to be localized to the cytoplasm, but following myogenesis, Myc-tagged Myo18B localized to some of the nuclei. Localization of endogenous Myo18B to the nucleus was observed in a subset of cultured rat ventricular myocytes, but Myo18B also localized to bands (A-bands) between Z-disks, labeled with anti-α-actinin-2 antibodies, in myofibrils. Notably, Myo18B did not appear to localize to the nucleus in ventricular myocytes exhibiting a prominent banding pattern.

Using anti-human MYO18B-N-terminus and anti-human MYO18B-C-terminus antibodies, Ajima et al. (2008) showed that Myo18B localized to actin stress fibers in differentiated C2C12 cells, but localization to the nucleus was not observed. Exogenously expressed MYO18B-GFP, but not GFP-tagged MYO18B lacking the N-terminal extension, similarly localized to actin fibers. Furthermore, the N-terminus alone was sufficient for localization to stress fibers. However, in frozen sections of mouse cardiac and skeletal muscle, immunofluorescence imaging indicated that Myo18B (green signal) localizes to Z-lines, labeled with anti-α-actinin antibodies (red signal). These findings contradict those of Salamon et al. (2003), who found that Myo18B localizes to the A-band, and we presume that the anti-Myo18B antibodies used by Ajima et al. exhibited poor specificity, at least in cardiac myocytes. The authors also generated *Myo18b* reporter knockout mice, but homozygous mutants died around embryonic day 10.5. LacZ (X-Gal) staining of heterozygous mutant embryos revealed that *Myo18b* is highly expressed in the heart, and also clearly in somites. Electron microscopy of embryonic day 10.5 hearts showed developing sarcomeres in wild-type hearts, whereas sarcomeric thick and thin filaments appeared less organized in homozygous mutant hearts, especially in cross-sections of developing myofibrils. Thus, Myo18B appears to localize to sarcomeres and may be critical for the formation and/or maintenance of myofibrils.

Consistent with a role in the heart, mutations of *MYO18B* have been associated with cardiomyopathies (Alazami et al., 2015; Malfatti et al., 2015; Mihaylova et al., 2020). The pathophysiology of the cardiomyopathies is unclear, although loss-of-function mutations of *myo18b* in zebrafish were reported to severely impair myofibrillogenesis in fast-twitch skeletal muscle cells (Berger et al., 2017; Gurung et al., 2017), suggesting that it may be explained by impaired formation and/or maintenance of myofibrils, as speculated by Ajima et al. (2008). Latham et al. (2020) provided insight into the function of Myo18B by showing that it initially localizes to nuclei during cardiac differentiation and then to actin stress fibers before incorporating into sarcomeres. Moreover, recombinant Myo18B heavy meromyosin exhibited negligible ATPase activity and failed to translocate F-actin filaments in *in vitro* gliding assays. These data suggest that Myo18B may be involved in sarcomere assembly in accord with the transition model in which myofibrils arise from actin stress fibers, which serve as premyofibrils and initially contain nonmuscle myosin IIB (Sanger et al., 2005; Sanger et al., 2010). Immunolabeling of Myo18B in cardiac myocytes, derived from human embryonic stem cells, together with anti-β-cardiac myosin (encoded by *MYH7*) or anti-α-actinin antibodies clearly showed that Myo18B localizes to sarcomeric A-bands, as distinct from Z-lines. The authors also confirmed that Myo18B binds actin filaments and proposed a model in which Myo18B tethers the thick filament to the thin (actin) filament and provides internal resistance to sarcomere length changes. Superresolution imaging by another group (Jiu et al., 2019) using U2OS cells suggested another function for Myo18B, which may also apply to sarcomeres, in which Myo18B mediates lateral stacking of nonmuscle myosin IIB-containing actin stress fibers, which notably are thought to be precursors of sarcomeres. Zhao et al. (2020) confirmed that Myo18B acts as “glue” for nonmuscle myosin two stacks. U2OS cells lacking Myo18B exhibited a paucity of thick ventral stress fibers and focal adhesions, as well as lower traction forces, when plated on a hard surface (glass), compared to control cells. Moreover, *MYO18B* knockout cells exhibited decreased migration velocity and directionality.

### Sequence alignments and predicted structure

Sequence alignments reveal moderate sequence identity and similarity between the shared motor, neck, and tail domains of Myo18A isoforms compared to Myo18B (Figure 1A). In contrast, the N- and C-termini are highly variable and exhibit low identity and similarity. One notable exception is the extreme N-terminus, particularly the first 19 amino acids, which exhibit high identity and similarity between Myo18Aγ and Myo18B (Figure 1B). The N-terminal extensions of Myo18Aα, Myo18Aγ, and Myo18B are distinctive features of class 18 myosins, and they are likely sufficient for localization to actin stress fibers in the case of Myo18Aα, or sarcomeric A-bands in the case of Myo18Aγ and Myo18B.

**FIGURE 2:**
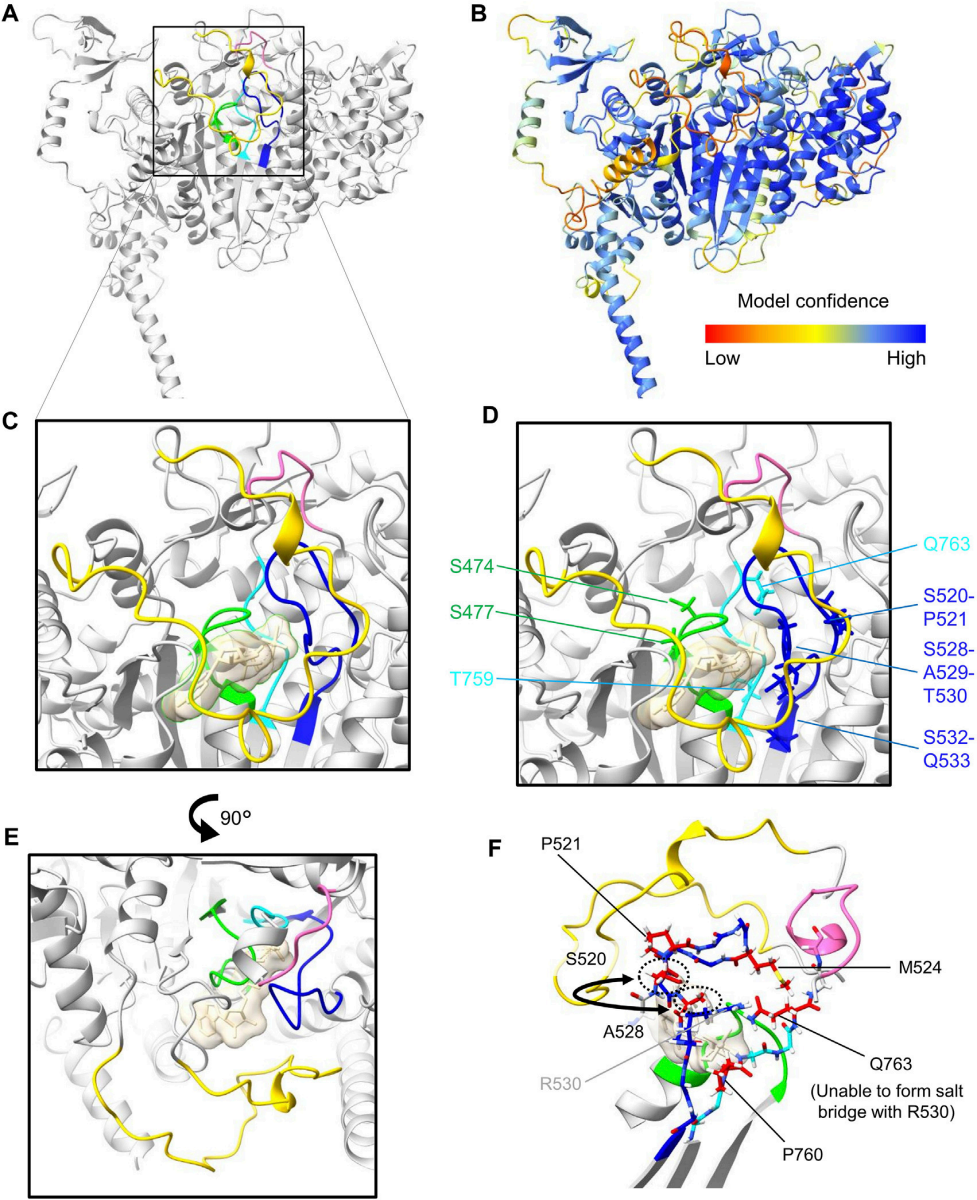
Protein structure prediction of (*Mus musculus*) Myo18Aγ. **(A)**, AlphaFold v2.3.2 was used to perform structure predictions via a Jupyter Notebook hosted on GitHub. Due to computational power constraints, predictions were restricted to amino acid residues 1 to 1400, encompassing the N-terminal extension, as well as the head domain. Visual representation of the structure was generated using ChimeraX. For clarity, only the head domain is represented (residues 281–1200). The ribbon representation of the head domain is shaded in gray, except for features forming and surrounding the nucleotide-binding pocket: the P-loop (G473–T480) is colored green, SW-1 (S520–Q533) is blue, and SW-2 (D758–Q763) is cyan. In addition, a small loop extension following SW-2 (G769–A774) is highlighted pink and the moderately large Myo18A-loop (E1024–L1052) is yellow. **(B)**, Ribbon representation of the head domain color-coded for the confidence score (pLDDT, predicted local distance difference test) of the model generated by AlphaFold. **(C)**, Close-up view of the nucleotide-binding pocket, albeit including the synthetic substrate ADP-AlF_4_ (transparent sand colored surface), incorporated by overlaying the structure 1MND from the PDB. **(D)**, Side chains of residues which deviate from censensus sequences are shown as sticks (see also Figure 1B). **(E)**, Rotated view (90° rotation relative to panels **(C, D)** highlighting the proximity of the moderately long Myo18A-loop (yellow) to the nucleotide-binding site. **(F)**, View optimized to show the side chains of residues within SW-1 and SW-2 which deviate (colored red) from the consensus sequences.

Guzik-Lendrum et al. (2013) previously highlighted significant deviations of Myo18A from conserved structural motifs of the nucleotide-binding pocket in the head domain of myosins, which include the P-loop (phosphate-binding loop), switch-1 (SW-1), and switch-2 (SW-2). Similar deviations can also be observed for Myo18B, as highlighted by Taft and Latham (2020) and depicted in Figures 1B, 2D. A model of the head domain of *Mus musculus* Myo18Aγ predicted using AlphaFold v2.3.2 (Jumper et al., 2021) is depicted in Figures 2A, B, including magnified views of the nucleotide-binding pocket (Figures 2C–F; PDB file available upon request). Note that a model of *Mus musculus* Myo18Aα was previously predicted and deposited on the beta version of the AlphaFold Protein Structure Database (accession number Q9JMH9). As expected, the prediction of the structure of the head domain of Myo18Aγ is very close to that of Myo18Aα (rmsd 0.557).

P-loops are recognized for their ability to bind both the phosphates of nucleotides and Mg^2+^ ions. Within myosins, the P-loop contains a highly conserved consensus sequence motif (GESGAGKT) (Ruppel and Spudich, 1996) (Figure 1B). Changes from this consensus sequence, such as substituting the negatively charged amino acid glutamic acid (E) with the uncharged polar amino acid serine (S) in Myo18A or the positively charged amino acid arginine (R) in Myo18B, may have a significant impact on ATP binding and hydrolysis.

Switch-1 is also a critical region in myosin proteins that undergoes considerable conformational changes upon nucleotide binding and hydrolysis (Smith and Rayment, 1996; Fujii and Namba, 2017; Kato et al., 2018). This was previously demonstrated through alanine scanning, which identified residues in switch-1 required for nucleotide binding and ATPase activity (Shimada et al., 1997). Both Myo18A and Myo18B harbor numerous deviations from the consensus sequence motif of switch-1 (Taft and Latham, 2020) (Figure 1B). Mapping of these deviations in our predicted model (Figure 2F) shows that hydrophobic residues replace charged and polar residues: (i) M524 replaces a positively charged residue typically seen in the variable position in other myosin sequences and (ii) A528 has effectively exchanged positions with S520, resulting in a deviation of the consensus sequence from (A520, S528) to (S520, A528). This swap, illustrated by the semicircular arrow (Figure 2F), positions A528 closer to the binding pocket.

Switch-2 is another important structural motif which has been deduced to stabilize the closed conformation of the nucleotide-binding pocket and thereby promote ATP hydrolysis (Furch et al., 1999; Trivedi et al., 2012). In both Myo18A and Myo18B, the position typically occupied by the hydrophobic amino acid isoleucine (I) in the switch-2 consensus sequence DIXGFE, where X denotes any residue, is instead filled by threonine (T). However, what is perhaps more remarkable, the presence of glutamine (Q763 in Figure 2F) instead of negatively charged glutamic acid at the end of the motif (DIXGFE → DTXGFQ) is expected to prevent formation of a salt bridge with a conserved arginine (R530 in Figure 2F) on switch-1 (Furch et al., 1999; Guzik-Lendrum et al., 2013), demonstrated to be critical for ATP hydrolysis and actin affinity (Onishi et al., 1997; Friedman et al., 1998; Onishi et al., 1998; Furch et al., 1999).

Other notable deviations from the consensus sequence in Myo18A are the presence of prolines (P) in switch-1 (P521) and switch-2 (P760) (Figures 2D, F), as well as the loop extensions E1024–L1052 (yellow in Figure 2), denoted the Myo18A-loop, and G769–A774 (pink in Figure 2). Myo18B also contains a proline in switch-2 (Figure 1B). These prolines may impair conformational flexibility of the loops required for catalysis. Loop extensions have previously been pointed out (Guzik-Lendrum et al., 2013). The large Myo18A-/Myo18B-loop (sequences shown in Figure 1B) is not predicted with high confidence, but as depicted in Figure 2E, the Myo18A-loop (colored yellow) is likely to hinder nucleotide access to the binding site. The small loop extension (colored pink) at the C-terminal end of switch-2 is predicted with high confidence and causes the whole loop to fold in a different conformation compared to known structures, such as myosin S1 fragments of *Dictyostelium discoideum* and *Bos taurus* (Protein Data Bank (PDB) identification codes 1MND and 8QYU, respectively).

## Conclusion

The genes encoding class 18 myosins, *Myo18a* and *Myo18b*, are highly expressed in the developing heart and somites, as well as in adult striated muscle, and deletion of either gene is embryonic lethal around embryonic day 11.5, notably at a time when the need for blood pumping function becomes vital for growth. Myo18B and specific isoforms of Myo18A may be involved in myogenesis, potentially playing roles in the transition of premyofibrils, nonmuscle myosin 2-containing stress fibers, to sarcomeres. In differentiated cardiac myocytes, both Myo18B and the gamma-isoform of Myo18A localize to the A-bands of sarcomeres, presumably via their respective N-terminal extensions (Figure 1A). Myo18Aγ and Myo18B most likely coassemble with class 2 myosins at low stoichiometric ratios to form mixed-class bipolar myosin filaments. They may function as scaffold proteins, potentially extending to the thin filaments to provide stability and internal resistance. These putative functions align with the apparent lack of ATPase activity in the head domains of class 18 myosins, although it remains to be established whether this holds true *in vivo*. Whether Myo18Aγ and Myo18B also regulate sarcomere function, for example, by acting as sarcomere length-dependent molecular brakes, remains to be explored.

Ultimately, Myo18Aγ and Myo18B are localized somewhere in the A-bands of highly organized sarcomeres, which have just been resolved at unprecedented resolution using cryo-electron microscopy by the groups of Roger Craig (Dutta et al., 2023) and Stefan Raunser (Tamborrini et al., 2023). Thus, there is a good chance that the precise arrangements and interaction partners of Myo18Aγ and Myo18B will soon be determined using such state-of-the-art structural data sets.

Assuming that class 18 myosins have evolved to become indispensible components of sarcomeres, the relative importance of PDZ-containing Myo18Aα remains to be clarified. Myo18Aα has been implicated in diverse functions, including Golgi structure and function and surfactant protein A binding, whereas the shorter PDZ-less isoform Myo18Aβ may act as a negative regulator via heterodimerization. One approach to assess the relative importance of the various Myo18A isoforms would be to genetically inhibit the expression of Myo18Aα in mice and screen for phenotypes. Subsequently, if no significant abnormalities manifest, the α-isoform-specific knockout mouse model could be further modified to additionally inhibit expression of the β-isoform, while retaining the expression of Myo18Aγ. That is, phenotypic analysis of Myo18A isoform-specific knockout mouse models may help to resolve the specific functions of the various isoforms.
